# Comparative analysis of multimodal biomarkers for amyloid-beta positivity detection in Alzheimer's disease cohorts

**DOI:** 10.3389/fnagi.2024.1345417

**Published:** 2024-02-26

**Authors:** Mostafa Mehdipour Ghazi, Per Selnes, Santiago Timón-Reina, Sandra Tecelão, Silvia Ingala, Atle Bjørnerud, Bjørn-Eivind Kirsebom, Tormod Fladby, Mads Nielsen

**Affiliations:** ^1^Department of Computer Science, Pioneer Centre for Artificial Intelligence, University of Copenhagen, Copenhagen, Denmark; ^2^Department of Neurology, Akershus University Hospital, Lørenskog, Norway; ^3^Institute of Clinical Medicine, Campus Ahus, University of Oslo, Oslo, Norway; ^4^Department of Radiology, Copenhagen University Hospital Rigshospitalet, Copenhagen, Denmark; ^5^Department of Physics, University of Oslo, Oslo, Norway; ^6^Unit for Computational Radiology and Artificial Intelligence, Oslo University Hospital, Oslo, Norway; ^7^Department of Neurology, University Hospital of North Norway, Tromsø, Norway; ^8^Department of Psychology, Faculty of Health Sciences, UiT The Arctic University of Norway, Tromsø, Norway

**Keywords:** Alzheimer's disease, amyloid-beta, magnetic resonance imaging, deep machine learning, biomarker classification

## Abstract

**Introduction:**

Efforts to develop cost-effective approaches for detecting amyloid pathology in Alzheimer's disease (AD) have gained significant momentum with a focus on biomarker classification. Recent research has explored non-invasive and readily accessible biomarkers, including magnetic resonance imaging (MRI) biomarkers and some AD risk factors.

**Methods:**

In this comprehensive study, we leveraged a diverse dataset, encompassing participants with varying cognitive statuses from multiple sources, including cohorts from the Alzheimer's Disease Neuroimaging Initiative (ADNI) and our in-house Dementia Disease Initiation (DDI) cohort. As brain amyloid plaques have been proposed as sufficient for AD diagnosis, our primary aim was to assess the effectiveness of multimodal biomarkers in identifying amyloid plaques, using deep machine learning methodologies.

**Results:**

Our findings underscore the robustness of the utilized methods in detecting amyloid beta positivity across multiple cohorts. Additionally, we investigated the potential of demographic data to enhance MRI-based amyloid detection. Notably, the inclusion of demographic risk factors significantly improved our models' ability to detect amyloid-beta positivity, particularly in early-stage cases, exemplified by an average area under the ROC curve of 0.836 in the unimpaired DDI cohort.

**Discussion:**

These promising, non-invasive, and cost-effective predictors of MRI biomarkers and demographic variables hold the potential for further refinement through considerations like APOE genotype and plasma markers.

## 1 Introduction

Alzheimer's disease (AD) is a neurodegenerative condition that leads to cognitive dysfunction and eventual dementia (Gaugler et al., 2022). The initial event in AD pathophysiology is the extracellular deposition of amyloid beta (Aβ) plaques in the brain, which can occur up to 20 years before the onset of dementia (Karran and De Strooper, 2022). This extended predementia phase offers a potential window for secondary prevention of AD dementia. However, defining the target population for such prevention strategies remains a lengthy, error-prone, and costly process (Scharre, 2019).

Aβ plaques are associated with longitudinal cognitive decline (Jack et al., 2013), and recently proposed guidelines (https://aaic.alz.org/diagnostic-criteria.asp) posit that they are sufficient to define AD. Research indicates that cognitive dysfunction in AD is closely linked to the deposition of intracellular tau neurofibrillary tangles (Braak and Braak, 1991). The combined impact of misfolded amyloid and tau proteins appears to trigger a cascade of events (Selkoe, 2001), that ultimately leads to dementia according to current disease models, albeit with varying timeframes. Aβ dysmetabolism leading to Aβ plaque deposition is followed by an extended pre-morbid and pre-dementia stage that may provide a window for intervention (Buchhave et al., 2012).

While amyloid and tau can be quantified through the analysis of cerebrospinal fluid (CSF) and positron emission tomography (PET) measures as well as plasma-based assays (Janelidze et al., 2016; Mattsson et al., 2016), defining neurodegeneration remains challenging. Current research criteria suggest that neurodegeneration can be measured using fluorodeoxyglucose (FDG) PET, magnetic resonance imaging (MRI), and CSF measurements (Schöll et al., 2019), but specific biomarkers and thresholds remain uncertain. These vague definitions, combined with the variable disease trajectories and clinical presentations, contribute to the heterogeneity of AD (Jack et al., 2013). Furthermore, the spatiotemporal relationships between AD biomarkers require further clarification, making the diagnostic and prognostic assessment of AD complex and necessitating a multimodal approach (Jack et al., 2018).

Several studies employing machine learning techniques to predict AD pathology have predominantly relied on single-cohort analysis with a limited number of multimodal biomarkers (Li et al., 2020; Tosun et al., 2021; Agostinho et al., 2022). Moreover, some investigations have strategically combined noninvasive and sensitive markers, including CSF measures and genetic data, with MRIs to underscore the effectiveness of integrating multimodal imaging and non-imaging markers for AD prediction, as demonstrated by Salvatore et al. (2015) and Moscoso et al. (2019). Despite these advancements, several limitations persist within the field in terms of data heterogeneity, constrained sample sizes, a limited number of cohorts, and the inherent challenges in harmonizing diverse datasets and biomarkers. Furthermore, the imperative for standardization across studies and validation in large, independent cohorts emerges as a critical necessity, ensuring that predictive models attain robust generalizability across varied populations and settings.

Considering the challenges inherent in AD pathology detection, our primary objective is the development of an artificial intelligence (AI) algorithm dedicated to predicting Aβ positivity and leveraging a comprehensive set of multimodal AD biomarkers in two distinct cohorts. Our study not only seeks to advance prediction model efficiency through novel machine learning methods but also holds the potential for cost reduction by leveraging noninvasive, readily available biomarkers. We rigorously validate this algorithm across multimodal biomarkers obtained from two independent non-demented cohorts, showcasing its robustness and applicability in diverse settings. The utilization of similar markers from these cohorts, matched based on analogous protocols and cutoffs, ensures compatibility and fairness in the acquired results. Additionally, our evaluation extends to the accuracy of models in predicting Aβ positivity by integrating noninvasive biomarkers, such as MRI measurements and demographic risk factors, with a specific emphasis on early-stage unimpaired cases.

## 2 Materials and methods

**Figure 1:**
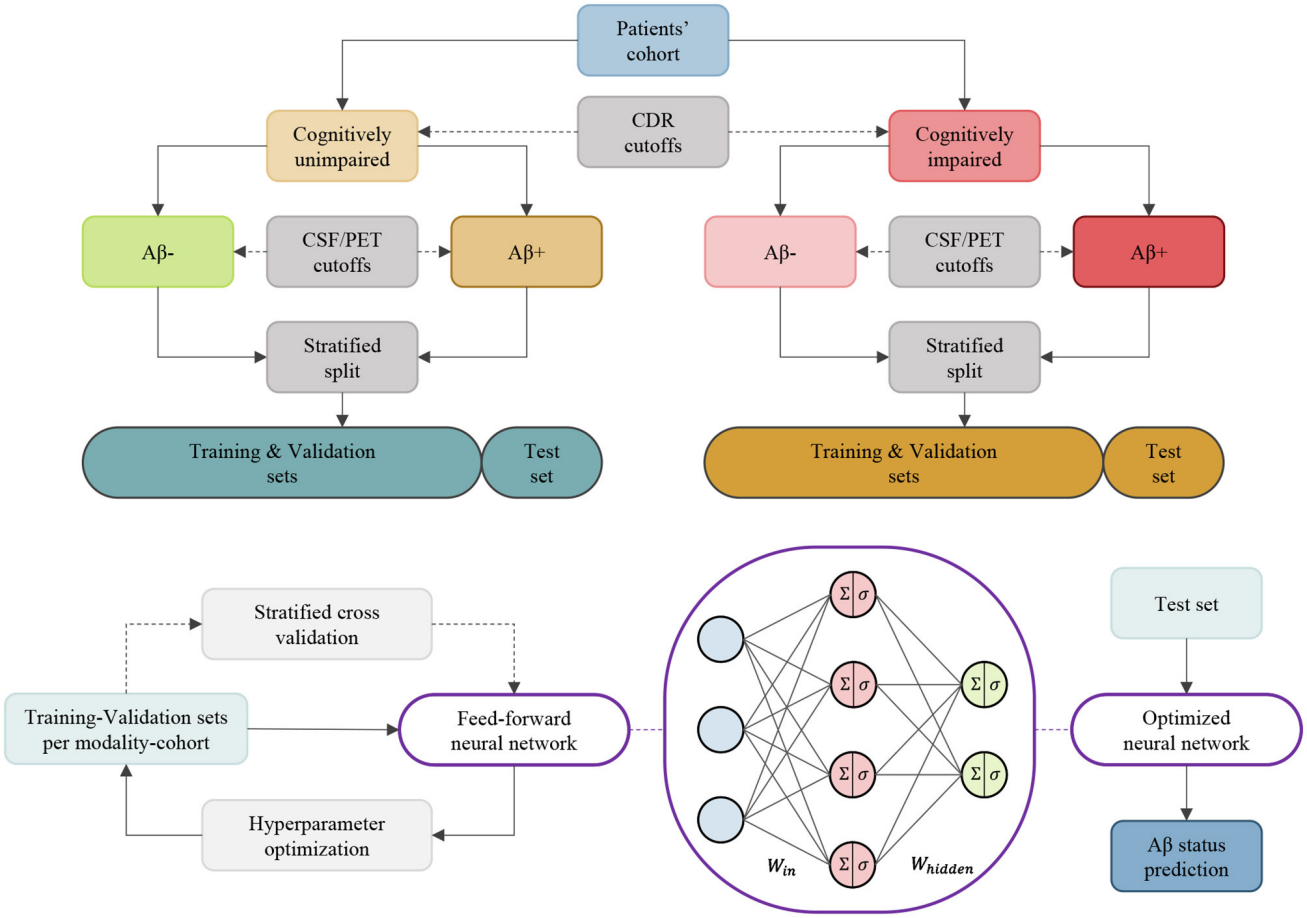
An overview of the methods employed for the processing and analysis of cohorts. These methods are independently applied to ADNI and DDI cohorts to obtain data subsets, and the training/testing is conducted per biomarker modality within each cohort/group.

This section outlines the data sources, processing steps, biomarkers, and the experimental and analytical procedures employed for amyloid-beta positivity classification. A summary of the methods employed in the processing and analysis of the cohorts is provided in Figure 1. The tools and codes utilized for data preparation and analysis are available at https://github.com/Mostafa-Ghazi/.

### 2.1 Study participants

We used data from two distinct cohorts for the analysis. The first cohort was sourced from publicly available data (Petersen et al., 2010) via the Alzheimer's Disease Neuroimaging Initiative (ADNI) website. The second cohort comprised participants from an in-house dataset (Fladby et al., 2017) from the Dementia Disease Initiation (DDI) study conducted in Norway. Tables 1, 2 show demographic details of the utilized cohorts per clinical diagnostic group.

**Table 1:** Demographics of the ADNI cohorts per clinical diagnostic group.

	**Cognitively unimpaired**		**Cognitively impaired**	
	**Aβ−**	**Aβ+**	**Aβ−**	**Aβ+**
# Subjects (male vs. female)	186/202	96/147	184/137	254/218
# Visits	795	423	576	717
APOE allele (ε−/ε+ ratio)	3.97	1.23	3.94	0.62
Age (years)	74.61 ± 7.26	77.15 ± 7.00	73.16 ± 8.65	75.38 ± 7.39
Education (years)	16.74 ± 2.50	16.42 ± 2.61	16.25 ± 2.66	16.03 ± 2.74
MMSE score	29.08 ± 1.20	28.80 ± 1.38	28.23 ± 1.98	26.84 ± 2.63

**Table 2:** Demographics of the DDI cohorts per clinical diagnostic group.

	**Cognitively unimpaired**		**Cognitively impaired**	
	**Aβ−**	**Aβ+**	**Aβ−**	**Aβ+**
# Subjects (male vs. female)	113/137	32/53	83/73	74/74
# Visits	333	108	222	191
APOE allele (ε−/ε+ ratio)	1.94	0.64	2.32	0.42
Age (years)	62.30 ± 9.53	68.21 ± 7.10	65.38 ± 9.06	70.47 ± 7.93
Education (years)	13.95 ± 2.88	14.18 ± 3.13	13.68 ± 3.31	13.23 ± 3.50
MMSE score	28.78 ± 1.76	28.73 ± 1.55	28.62 ± 1.73	27.49 ± 2.72

#### 2.1.1 ADNI

The ADNI was launched in 2003 as a public-private partnership, led by principal investigator Michael W. Weiner, MD. The primary goal of ADNI has been to test whether serial MRI, PET, other biological markers, and clinical and neuropsychological assessment can be combined to measure the progression of mild cognitive impairment (MCI) and early Alzheimer's disease. We included all participants from the ADNI-MERGE (1/GO/2/3) cohorts with available clinical dementia rating (CDR) values and Aβ status. This led to a dataset comprising 1,218 unimpaired and 1,293 impaired patients with missing modality biomarkers from the ADNI cohort. Refer to Table 1 for demographic details.

#### 2.1.2 DDI

The multi-site DDI cohort study comprised at-risk and clinical cases with subjective cognitive decline (SCD) (Jessen et al., 2014) and MCI (Albert et al., 2011) who had standardized MRI and CSF measurements (Fladby et al., 2017; Siafarikas et al., 2021). In addition, healthy controls (HC) were included from spouses of patients and from patients who completed lumbar punctures in connection with orthopedic surgery. For both cases and controls, MRI scans, neuropsychological assessments, and samples of plasma and CSF were collected within 3 months of inclusion. Ethical approval for the study was obtained from the Regional Committees for Medical and Health Research Ethics (REK), ensuring compliance with ethical standards. All participants provided written informed consent, and all research procedures adhered to the relevant REK guidelines and the principles outlined in the Declaration of Helsinki. We included all participants from the DDI cohorts with available CDR values and Aβ status. This led to a dataset comprising 441 unimpaired and 413 impaired patients with missing modality biomarkers from the DDI cohort. Refer to Table 2 for demographic details.

### 2.2 Study biomarkers

**Figure 2:**
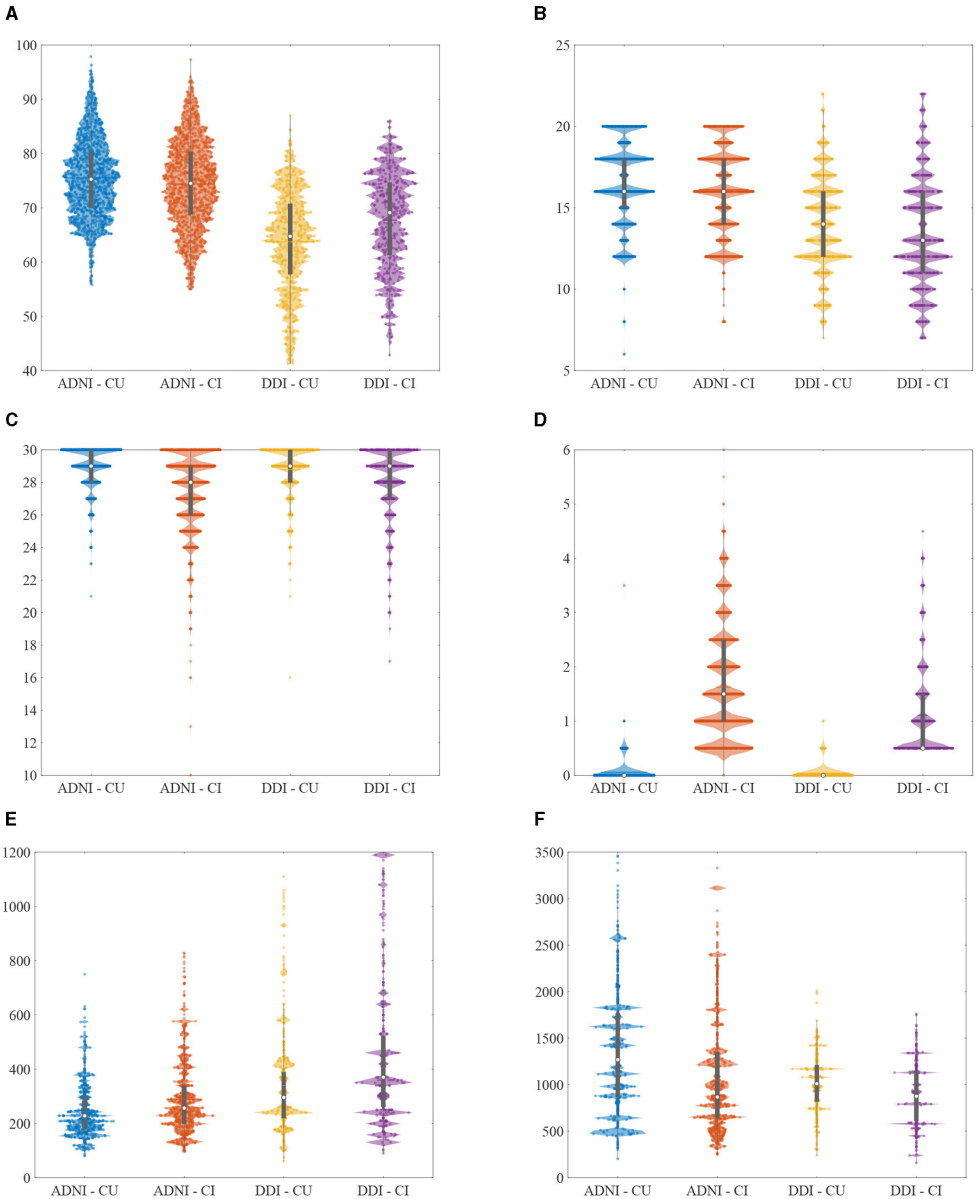
Violin plots of the selected non-imaging AD biomarkers utilized in this study from different cohorts. Note that clipped values of Aβ42 (>1,700 or < 200) were replaced with the recalculated results. **(A)** Age (years). **(B)** Education (years). **(C)** MMSE (scores). **(D)** CDR-SB (scores). **(E)** T-tau (pg/mL). **(F)** Aβ42 (pg/mL).

**Figure 3:**
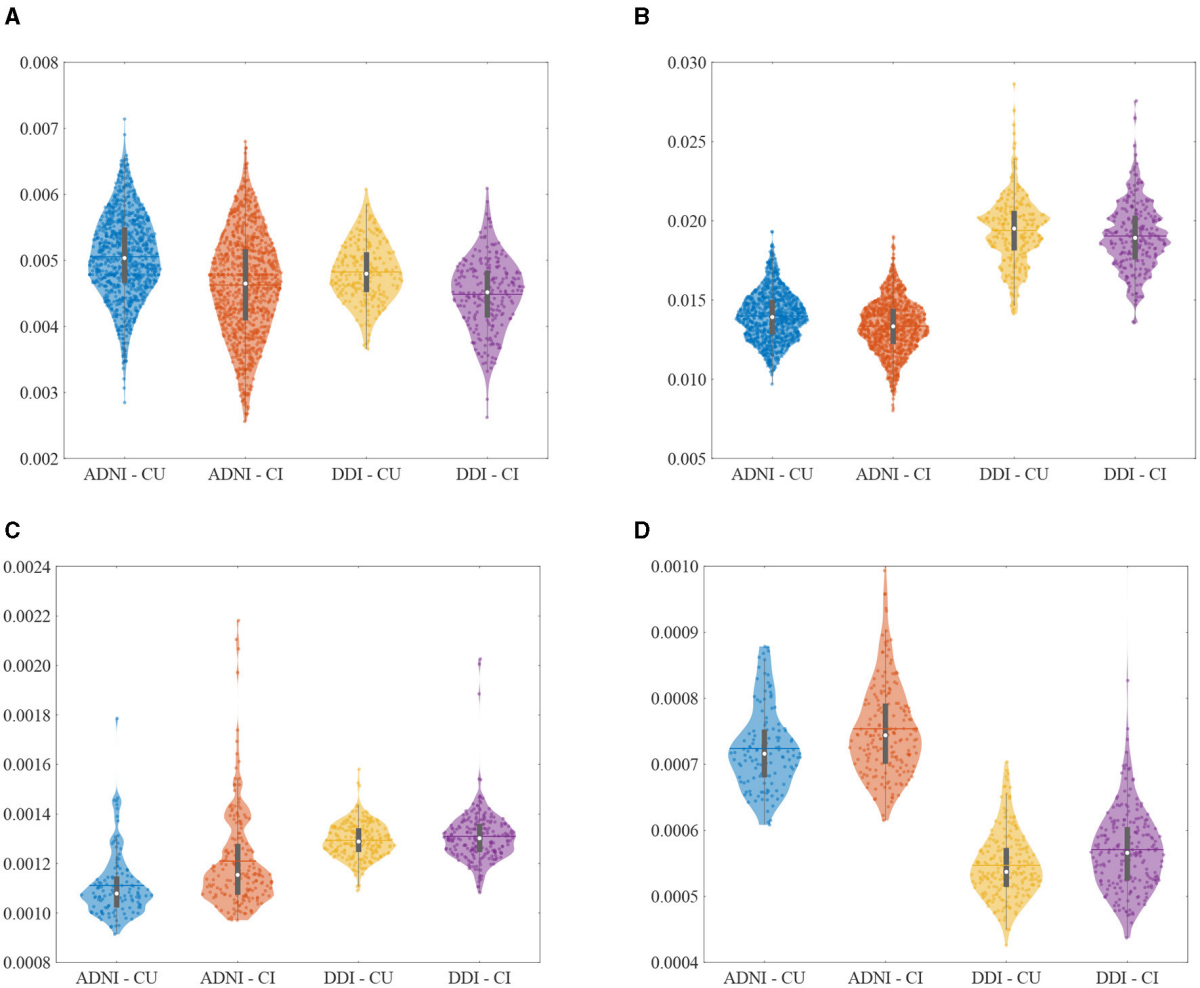
Violin plots of the selected imaging AD biomarkers utilized in this study from different cohorts. **(A)** Hippocampus volume (normalized). **(B)** Middle temporal gyrus (normalized). **(C)** Uncinate fasciculus (AD). **(D)** Inf fronto-occipital fasciculus (RD).

We used multimodal biomarkers to study Aβ positivity of different cohorts. These markers were selected from demographic risk factors, cognitive scores, and CSF and imaging (MRI/PET) measurements. Figures 2, 3 illustrate the distribution of some selected key multimodal AD biomarkers utilized in this study from the two datasets.

#### 2.2.1 Risk factors

To capture and assess risk factors, we selected the demographic variables of age, sex, and years of education as key determinants within both the ADNI and DDI cohorts.

#### 2.2.2 Cognitive scores

In both the ADNI and DDI cohorts, we employed scores from the Mini-Mental State Examination (MMSE) and the Clinical Dementia Rating-Sum of Boxes (CDR-SB). Furthermore, we utilized the Alzheimer's Disease Assessment Scale Cognitive (ADAS-Cog) with 13 items from the ADNI dataset and the learning subscale of the Consortium to Establish a Registry for Alzheimer's Disease (CERAD-Learning) from the DDI dataset. The classification of individuals into cognitively unimpaired (CU) or impaired (CI) categories was based on CDR values, where a CDR score of 0 indicated unimpaired status, and a score of 0.5 signified impairment (Morris et al., 2001; Aisen et al., 2010).

#### 2.2.3 CSF and PET measures

Aβ42, Aβ40, phosphorylated tau (p-tau181), total tau (t-tau), Neurogranin (Ng), and β-site amyloid-precursor-protein cleaving enzyme 1 (BACE1) were partially quantified in the CSF samples collected from both the ADNI and DDI cohorts. Pathological Aβ levels in the ADNI dataset were determined by applying a threshold of 1.11 to the radiotracer florbetapir (18F-AV-45) in PET scans without using CSF biomarkers, as previously validated by Royse et al. (2021). More specifically, we categorized patients with 18F-AV-45 values ≥1.11 into the Aβ+ group, while those with values below this threshold were assigned to the Aβ− group. In the DDI dataset, we established Aβ42/40 ratio cutoffs with a range of 0.077, derived from receiver operating curve (ROC) analysis using visual read results of Flutemetamol (18F-Flut) PET scans as the standard reference (Siafarikas et al., 2021). Aβ measurements were excluded from predictor variables in the model, as they were partially used for status definition in DDI and represent the same features as measured with PET in ADNI. Note that we achieved an area under the ROC curve of 0.98 when fitting Aβ42 values using decision trees for binary 18F-AV-45 classification. Moreover, since there were not enough ADNI samples with available Ng and BACE1 measurements, we only used p-tau181 and t-tau variables for the classification purpose in ADNI.

#### 2.2.4 MRI measures

MRI scans were obtained on multi-vendor systems including Siemens, Philips, and GE, at field strengths of 1.5 and 3 T. The T1-weighted images were used for volumetric analysis with slice thicknesses from 1 to 2 mm, while diffusion-weighted imaging (DWI) series were used for diffusion tensor-based analysis.

T1-weighted volumetric assessments were conducted using distinct segmentation tools: FreeSurfer (Fischl et al., 2002) in the ADNI cohort and the FAST-AID Brain (Mehdipour Ghazi and Nielsen, 2022) in the DDI cohort. Regional volumes were computed based on the segmentation results obtained from T1-weighted brain MRI scans. Subsequently, these estimated volumes were adjusted for the total intracranial volume (ICV). In the ADNI dataset, the analysis encompassed 6 brain regions, comprising the ventricles, hippocampus, entorhinal cortex, fusiform gyrus, middle temporal gyrus, and the whole brain. Conversely, the DDI cohort examined a more extensive set of 68 regions, encompassing both left and right compartments of the 132 segmented areas combined.

DWI scans underwent rigorous analysis using the FSL tool (Smith et al., 2004). To ensure data accuracy, corrections were applied to mitigate Eddy-current distortion and head motion, taking into consideration the available b0 scan. Subsequently, a diffusion tensor was modeled at every voxel within the brain, utilizing the FSL fit function on the corrected DWI scans. Scalar anisotropy and diffusivity maps were derived from the eigenvalues of the diffusion tensor, yielding fractional anisotropy (FA), axial diffusivity (AD), radial diffusivity (RD), and mean diffusivity (MD). To unveil spatial patterns of interest, a voxel-wise statistical analysis of the DTI maps was executed via the TBSS function (Smith et al., 2006). The corrected FA images were then registered to the white-matter tractography atlas sourced from Johns Hopkins University (JHU) (Hua et al., 2008). This process resulted in the calculation of 20 and 57 regions of interest (ROIs) for each anisotropy and diffusivity feature within the DDI and ADNI datasets, respectively.

### 2.3 Statistical analysis

The statistical analysis involved the utilization of appropriate tests based on variable type and group comparisons. For continuous variables within both the impaired and unimpaired cohorts, the nonparametric Wilcoxon rank-sum test was employed to assess statistical differences between the two groups (Aβ±). Categorical variables, on the other hand, were subject to Fisher's exact test when dealing with binary outcomes, and the Chi-squares test when analyzing groups with nonbinary values. A significance threshold of *p* < 0.05 was employed to guide the determination of hypothesis test outcomes.

### 2.4 Amyloid-beta status prediction

Our approach for Aβ± classification involved the utilization of feedforward artificial neural networks comprising two fully connected layers with learnable parameters and nonlinear activation functions. These networks were employed to predict the Aβ status by leveraging multivariate biomarkers from each modality, both individually and in combination. These networks underwent supervised training, during which they learned to encode high-dimensional input data, abstracting latent variables while disregarding insignificant information and minimizing classification errors through an output layer.

To optimize the network's performance and ensure robustness, we conducted a rigorous training and inference process. We randomly partitioned each of the two datasets into training (80%) and testing (20%) subsets using a stratified approach. We applied a five-fold stratified cross-validation and testing procedure to the training and test data to tune the network's hyperparameters, allowing at most 1,000 iterations, and to assess the generalization capability.

We assessed the performance of our prediction models by employing key metrics including total accuracy, the area under the ROC curve (AUC), precision, recall (sensitivity), and specificity. These metrics were consistently evaluated across various cross-validation folds applied to the test sets. This approach allows for a comprehensive interpretation of results, effectively addressing concerns related to data size, biases, and classification errors of any kind.

## 3 Results

In the ADNI cohorts considering both impaired and unimpaired individuals, several key variables exhibited statistically significant differences between the Aβ± groups. These variables included age, ADAS-Cog, MMSE, p-tau, t-tau, Aβ42, as well as the volumetric measurements of ventricles, hippocampus, whole brain, and 47 regional diffusion parameters. Similarly, within the DDI cohorts comprising both impaired and unimpaired individuals, the Aβ± groups demonstrated notable disparities in various variables. Specifically, age, CERAD-Learning, p-tau, t-tau, BACE1, Ng, volumes of seven distinct brain regions, and two regional diffusion measures displayed statistically significant differences between the two Aβ groups. **Figure 5** depicts a visual representation of the statistical analysis conducted on the ADNI biomarkers.

Within the impaired ADNI cohort, significant differences between the Aβ± groups were observed in CDR-SB, volumetric measurements of the entorhinal, fusiform, and middle temporal gyrus, as well as seven regional diffusion metrics. Conversely, in the unimpaired ADNI cohort, distinctions emerged in terms of sex, years of education, and 136 regional diffusion measures. Besides, in the impaired DDI cohort, Aβ± group disparities were evident in CDR-SB and MMSE scores, volumes spanning 33 distinct brain regions, and two regional diffusion metrics. However, in the unimpaired DDI cohort, significant differences were only noted in terms of sex and a regional diffusion measure. **Figure 6** presents a visual representation of the statistical analysis conducted on the DDI biomarkers.

Tables 3–6 provide a comprehensive summary of the Aβ status classification outcomes obtained from the test sets within the ADNI and DDI cohorts, covering both the impaired and unimpaired groups. The results suggest that CSF measures exhibit the highest accuracy, with imaging markers and cognitive scores following behind. Additionally, the minimal fluctuations observed around the average accuracies show the robustness of the models across various cross-validation folds. Furthermore, the congruence in results between the ADNI and DDI cohorts underscores the generalizability of these models.

**Table 3:** Aβ status classification results (five-fold mean ± SD) for the unimpaired group of the test ADNI.

**Biomarker**	**# Samples**	**AUC**	**Accuracy**	**Sensitivity**	**Specificity**	**Precision**
Cognitive	243	0.603 ± 0.004	0.597 ± 0.005	0.500 ± 0.017	0.648 ± 0.006	0.429 ± 0.007
CSF	114	0.862 ± 0.007	0.802 ± 0.008	0.784 ± 0.019	0.810 ± 0.015	0.666 ± 0.015
T1-MRI	187	0.651 ± 0.012	0.634 ± 0.008	0.512 ± 0.058	0.694 ± 0.028	0.447 ± 0.012
Diffusion-MRI	24	0.825 ± 0.049	0.775 ± 0.037	0.514 ± 0.163	0.882 ± 0.042	0.644 ± 0.057
T1-MRI and risk	187	0.720 ± 0.013	0.726 ± 0.010	0.489 ± 0.058	0.841 ± 0.022	0.599 ± 0.018

**Table 4:** Aβ status classification results (five-fold mean ± SD) for the impaired group of the test ADNI.

**Biomarker**	**# Samples**	**AUC**	**Accuracy**	**Sensitivity**	**Specificity**	**Precision**
Cognitive	258	0.760 ± 0.001	0.709 ± 0.000	0.632 ± 0.004	0.805 ± 0.005	0.801 ± 0.003
CSF	155	0.925 ± 0.003	0.849 ± 0.013	0.873 ± 0.024	0.816 ± 0.007	0.871 ± 0.004
T1-MRI	194	0.753 ± 0.002	0.697 ± 0.004	0.737 ± 0.011	0.649 ± 0.017	0.713 ± 0.008
Diffusion-MRI	37	0.828 ± 0.027	0.735 ± 0.048	0.760 ± 0.022	0.706 ± 0.083	0.755 ± 0.059
T1-MRI and risk	194	0.862 ± 0.003	0.777 ± 0.024	0.792 ± 0.021	0.760 ± 0.043	0.796 ± 0.030

**Table 5:** Aβ status classification results (five-fold mean ± SD) for the unimpaired group of the test DDI.

**Biomarker**	**# Samples**	**AUC**	**Accuracy**	**Sensitivity**	**Specificity**	**Precision**
Cognitive	88	0.590 ± 0.008	0.602 ± 0.043	0.514 ± 0.052	0.630 ± 0.070	0.306 ± 0.025
CSF	80	0.843 ± 0.016	0.858 ± 0.019	0.670 ± 0.027	0.920 ± 0.022	0.739 ± 0.057
T1-MRI	48	0.674 ± 0.010	0.671 ± 0.040	0.415 ± 0.042	0.766 ± 0.055	0.402 ± 0.060
Diffusion-MRI	44	0.753 ± 0.008	0.809 ± 0.026	0.517 ± 0.109	0.919 ± 0.036	0.716 ± 0.079
T1-MRI and risk	48	0.836 ± 0.015	0.746 ± 0.040	0.569 ± 0.129	0.811 ± 0.033	0.526 ± 0.063

**Table 6:** Aβ status classification results (five-fold mean ± SD) for the impaired group of the test DDI.

**Biomarker**	**# Samples**	**AUC**	**Accuracy**	**Sensitivity**	**Specificity**	**Precision**
Cognitive	82	0.732 ± 0.007	0.654 ± 0.014	0.658 ± 0.065	0.650 ± 0.061	0.621 ± 0.026
CSF	69	0.910 ± 0.003	0.829 ± 0.019	0.820 ± 0.018	0.840 ± 0.029	0.794 ± 0.030
T1-MRI	44	0.785 ± 0.029	0.736 ± 0.057	0.756 ± 0.101	0.723 ± 0.032	0.652 ± 0.052
Diffusion-MRI	43	0.682 ± 0.012	0.637 ± 0.027	0.553 ± 0.067	0.692 ± 0.000	0.539 ± 0.030
T1-MRI and risk	44	0.805 ± 0.044	0.723 ± 0.059	0.678 ± 0.107	0.754 ± 0.044	0.654 ± 0.062

**Figure 4:**
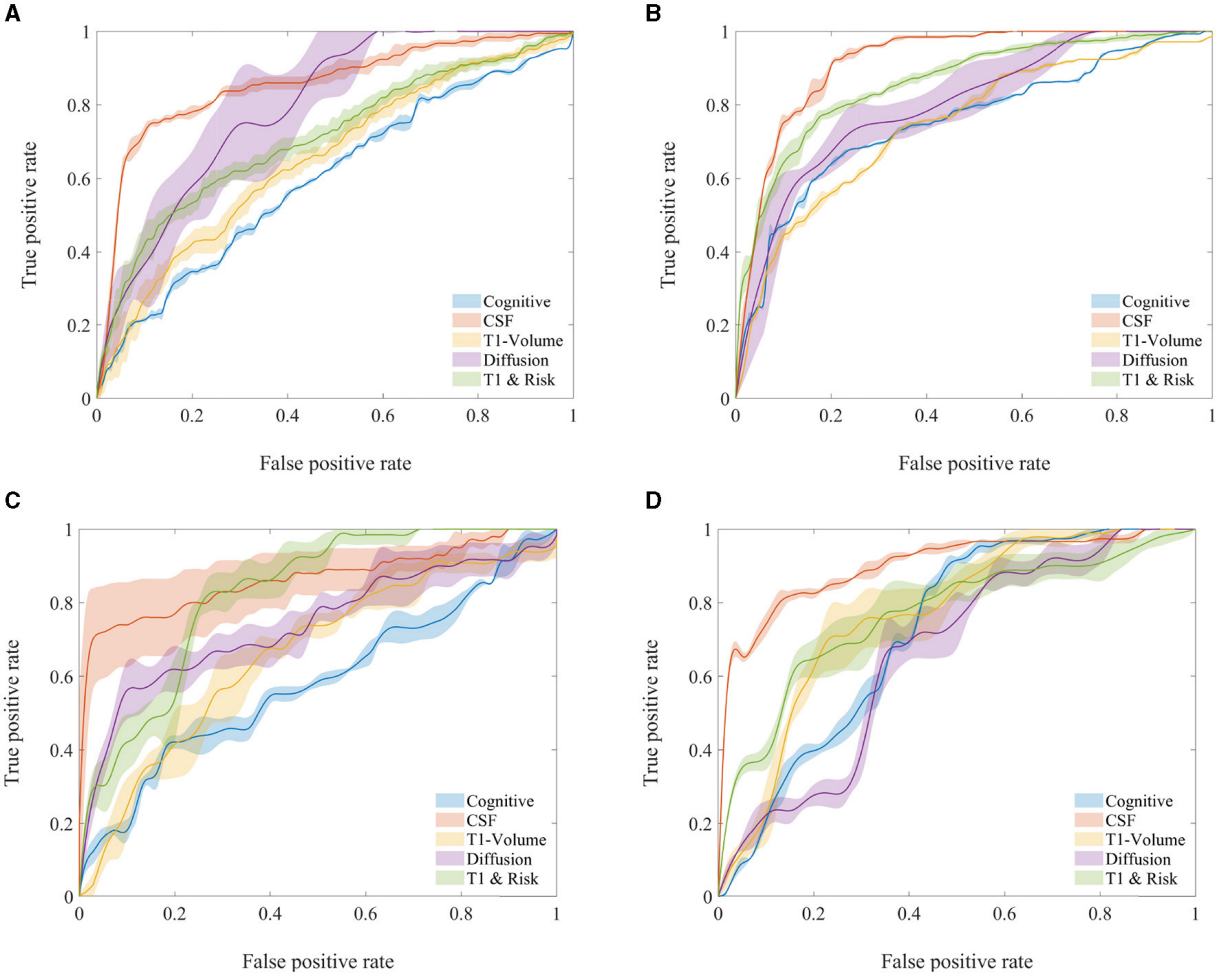
Average ROC curves and their associated 95% confidence intervals for various AD biomarker modalities of the test cohorts used in Aβ status classification. **(A)** ADNI—unimpaired. **(B)** ADNI—impaired. **(C)** DDI—unimpaired. **(D)** DDI—impaired.

To complement the result interpretation, we have additionally provided visual representations of the ROC curves in Figure 4 for all cohorts within the utilized test sets. The observed variability in the diffusion-MRI measurements can be attributed to the limited availability of data points in this specific modality. Furthermore, the lower and fluctuating accuracy of CSF measurements in the unimpaired DDI cohort, compared to alternative modalities and cohorts, may be attributed to slight differences in the CSF measurement protocols between DDI and ADNI, potentially influencing the detection of Aβ positivity in the unimpaired groups. It is also worth noting that the higher accuracies achieved by the T1-MRI measures in the DDI cohorts can be primarily attributed to the utilization of a larger set of robust regional volumes obtained through FAST-AID Brain.

## 4 Discussion

This study leveraged two distinct cohorts, ADNI and DDI, characterized by nearly the same sets of biomarkers, ensuring a fair and comprehensive analysis. When comparing the clinical-demographic differences between the ADNI and DDI cohorts, patients in the ADNI cohort tend to be older than those in the DDI cohort, yet they possess a higher number of education years. Additionally, the ADNI cohort exhibits a lower percentage of cases with APOE+ alleles compared to the DDI cohort. The distribution of subjects by sex is nearly equivalent in both ADNI and DDI, with a higher proportion of females in the unimpaired cohort and a greater prevalence of males in the impaired cohort.

The prediction results obtained in this study reveal consistent accuracies across both the ADNI and DDI cohorts for Aβ status classification using deep learning models. Notably, CSF markers yielded the highest accuracy, followed by imaging biomarkers and cognitive scores. This pattern aligns with previous research, including the hypothetical model proposed by Jack et al. (2010) and the latest studies on multimodal biomarkers of AD (Tosun et al., 2021). The consistent findings underscore the robustness and generalizability of our models in achieving these accuracies.

The high AUCs obtained using non-amyloid CSF variables for the unimpaired and impaired groups implicate amyloid-related neurodegeneration both at early stages and during AD progression (Kirsebom et al., 2018, 2022). Besides, our classification results were more favorable in the impaired cohorts, attributed to the presence of pronounced biomarker changes and cognitive impairment developments in these groups. This trend mirrors earlier findings in the literature (Tosun et al., 2021), highlighting the relative challenges of Aβ status classification within the cognitively normal populations.

The high AUCs for diffusion-weighted and T1-weighted MRIs are in accordance with early changes in diffusivity measures and later neurodegenerative changes (Selnes et al., 2013). In the ADNI cohorts, the limited size of diffusion-weighted MRI data precludes a definitive conclusion regarding their performance compared to T1-weighted MRIs. In the DDI cohort, diffusion-weighted MRI markers exhibit higher accuracy in predicting Aβ positivity among early AD patients within the unimpaired cohort. Still, the observed discrepancy in results is greater compared to T1-weighted MRIs.

In general, cognitive markers exhibit lower predictive power for AD compared to imaging and CSF markers, aligning with findings in the existing literature discussed in various hypothetical, statistical, and learning models (Jack et al., 2013; Mehdipour Ghazi et al., 2019, 2021). However, it is noteworthy that certain cognitive or neuropsychological biomarkers, such as auditory-verbal assessments, have demonstrated substantial efficacy in detecting AD in the early stages (Zandifar et al., 2020; Mehdipour Ghazi et al., 2021).

**Figure 5:**
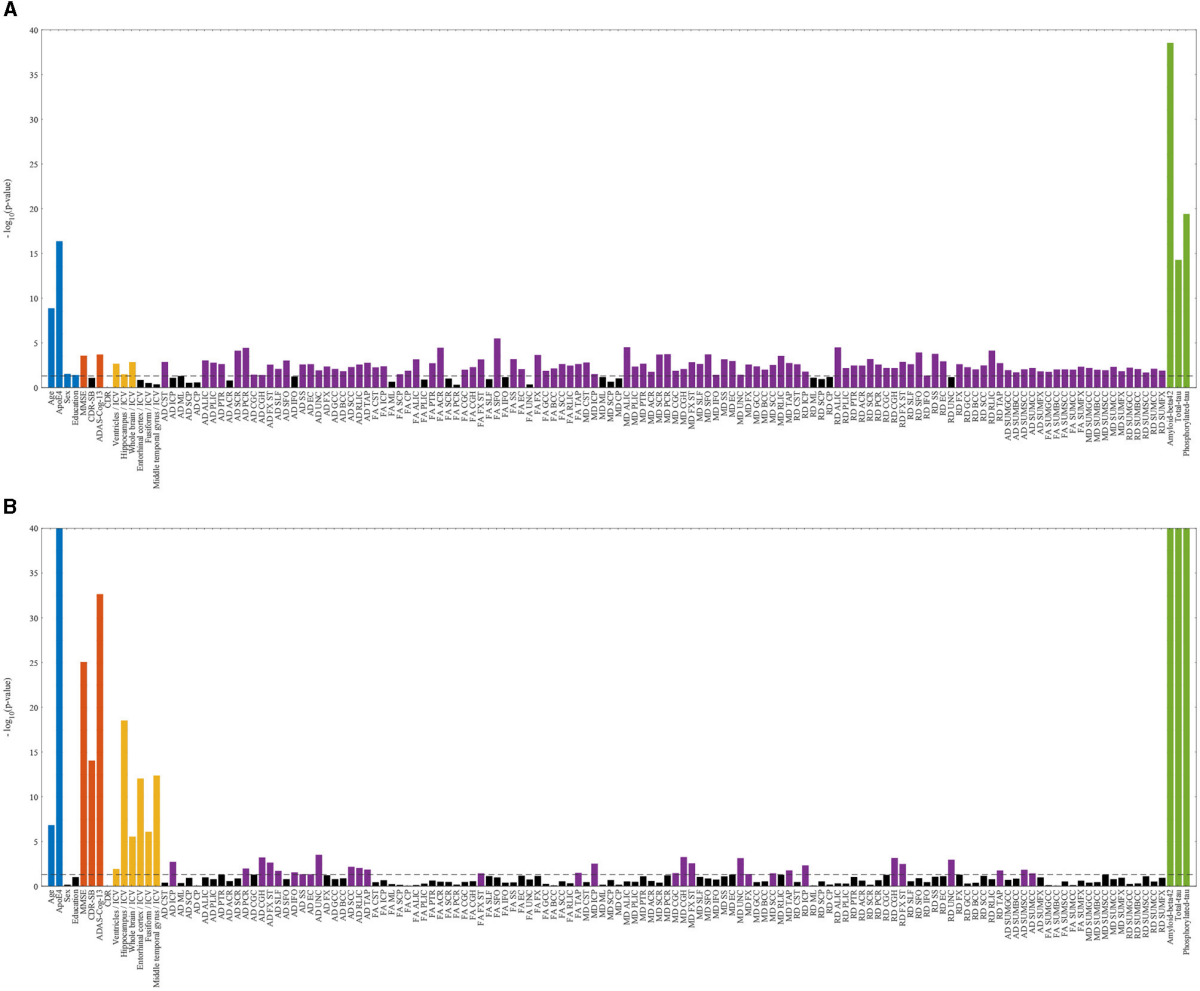
Statistically significant differences between Aβ± groups of ADNI cohorts with logarithmic *p*-values per biomarker. The dashed line indicates the 0.05 threshold, while non-significant markers are represented by black bars. Statistically significant risk factors (blue), cognitive scores (red), T1-weighted MRIs (yellow), diffusion-weighted MRIs (violet), and CSF markers (green) are highlighted. **(A)** ADNI—unimpaired. **(B)** ADNI—impaired.

**Figure 6:**
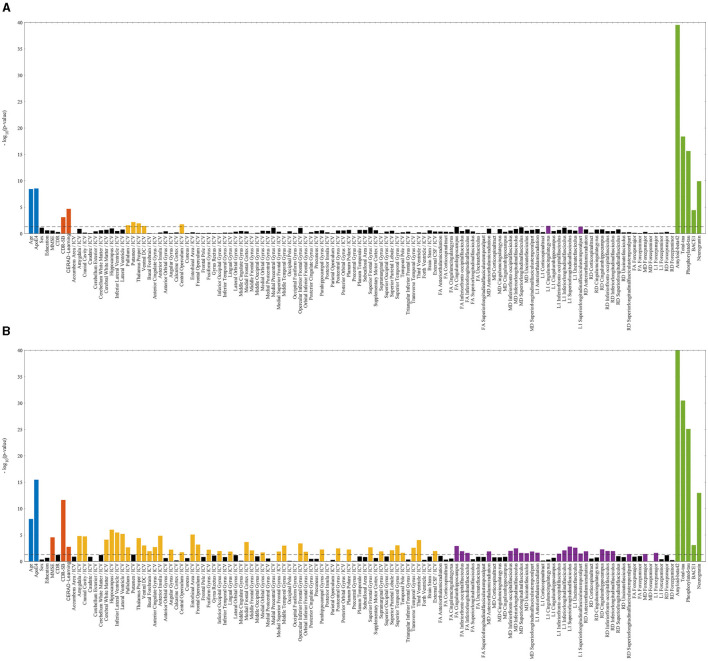
Statistically significant differences between Aβ± groups of DDI cohorts with logarithmic *p*-values per biomarker. The dashed line indicates the 0.05 threshold, while non-significant markers are represented by black bars. Statistically significant risk factors (blue), cognitive scores (red), T1-weighted MRIs (yellow), diffusion-weighted MRIs (violet), and CSF markers (green) are highlighted. **(A)** DDI—unimpaired. **(B)** DDI—impaired.

The results from the statistical analyses presented in Figures 5, 6 reveal statistically significant differences in some demographic risk factors and all CSF measures between Aβ± groups across all cases. Additionally, as cohorts transition to impairment, various biomarkers, including cognitive scores and MRI measurements, demonstrate significance. Notably, T1-weighted MRIs are particularly emphasized in the impaired DDI cohort, benefiting from a detailed regional analysis provided by FAST-AID Brain. Conversely, diffusion-weighted MRIs exhibit prominence in the unimpaired ADNI cohort, possibly attributed to the utilization of a more extensive set of regional markers and a smaller dataset from ADNI.

Furthermore, the combination of MRI measurements with demographic risk factors demonstrated an enhancement in classification performance across different scenarios. The literature has previously demonstrated the enhanced accuracy achieved through the concatenation of MRI measurements with other risk factors (Ten Kate et al., 2018; Tosun et al., 2021). Particularly intriguing is the observation that this improvement was more pronounced in the unimpaired cohorts. This outcome bears significant implications for early AD detection using noninvasive biomarkers, where early and accurate identification within this subset of individuals holds particular importance.

Finally, the utilized noninvasive and cost-effective predictors involving MRI biomarkers and demographic variables show promise for refinement through additional considerations, such as the APOE genotype and plasma markers. However, it is essential to acknowledge that utilizing the APOE genotype introduces challenges, mainly rooted in ethical considerations, privacy concerns, and the sensitivity of genetic information. On the contrary, plasma markers pose challenges due to their limited establishment in AD research, potentially hindered by a lack of substantial representation in large cohorts alongside other markers for comprehensive analysis.

## Data availability statement

The data analyzed in this study is subject to the following licenses/restrictions. The public ADNI datasets utilized in this study are accessible via https://adni.loni.usc.edu/data-samples/access-data/. However, the in-house DDI datasets used in this article are not readily available due to ethical and patient privacy restrictions. Requests to access these datasets should be directed at: TF (tormod.fladby@medisin.uio.no).

## Ethics statement

Ethical approval was not required for the study involving humans in accordance with the local legislation and institutional requirements. Written informed consent to participate in this study was not required from the participants or the participants' legal guardians/next of kin in accordance with the national legislation and the institutional requirements.

## Author contributions

MM: Conceptualization, Formal analysis, Investigation, Methodology, Software, Validation, Visualization, Writing—original draft. PS: Conceptualization, Investigation, Project administration, Supervision, Validation, Writing—review & editing. ST-R: Data curation, Resources, Writing—review & editing. ST: Resources, Writing—review & editing. SI: Writing—review & editing. AB: Writing—review & editing. B-EK: Resources, Writing—review & editing. TF: Funding acquisition, Project administration, Supervision, Writing—review & editing. MN: Funding acquisition, Project administration, Supervision, Writing—review & editing.
